# Multicaloric effect in a multiferroic composite of Gd_5_(Si,Ge)_4_ microparticles embedded into a ferroelectric PVDF matrix

**DOI:** 10.1038/s41598-019-54635-8

**Published:** 2019-12-04

**Authors:** V. M. Andrade, A. Amirov, D. Yusupov, B. Pimentel, N. Barroca, A. L. Pires, J. H. Belo, A. M. Pereira, M. A. Valente, J. P. Araújo, M. S. Reis

**Affiliations:** 1 IFIMUP and IN-Institute of Nanoscience and Nanotechnology, Physics and Astronomy Department of Science Faculty, University of Porto, Rua do Campo Alegre, 687, 4169-007 Porto, Portugal; 2 ‘Gleb Wataghin’ Physics Institute, State University of Campinas (UNICAMP), C.P. 6165, 13.083-970 Campinas, S.P. Brazil; 3 Laboratory of Novel Magnetic Materials & Institute of Physics Mathematics and Informational Technologies, Immanuel Kant Baltic Federal University, Kaliningrad, Russia; 4 Amirkhanov Institute of Physics Daghestan Scientific Center, Russian Academy of Sciences, Makhachkala, Russia; 5 Physics Institute, Fluminense Federal University, Av. Gal. Milton Tavares de Souza s/n, 24210-346 Niterói-RJ, Brazil; 6 Department of Physics and I3N, University of Aveiro, 3810-193 Aveiro, Portugal

**Keywords:** Materials science, Ferroelectrics and multiferroics, Ferromagnetism, Magnetic properties and materials, Surfaces, interfaces and thin films

## Abstract

The coupling between electric, magnetic and elastic features in multiferroic materials is an emerging field in materials science, with important applications on alternative solid-state cooling technologies, energy harvesting and sensors/actuators. In this direction, we developed a thorough investigation of a multiferroic composite, comprising magnetocaloric/magnetostrictive Gd${}_{5}$Si${}_{2.4}$Ge${}_{1.6}$ microparticles blended into a piezo- and pyroelectric poly(vinylidene) fluoride (PVDF) matrix. Using a simple solvent casting technique, the formation and stabilization of PVDF electroactive phases are improved when the filler content increases from 2 to 12 weight fraction (wt.%). This effect greatly contributes to the magnetoelectric (ME) coupling, with the ME coefficient ${\alpha }_{ME}$ increasing from 0.3 V/cm.Oe to 2.2 V/cm.Oe, by increasing the amount of magnetic material. In addition, magnetic measurements revealed that the ME-coupling has influenced the magnetocaloric effect via a contribution from the electroactive polymer and hence leading to a multicaloric effect. These results contribute to the development of multifunctional systems for novel technologies.

## Introduction

Recently, efforts to find materials simultaneously presenting more than one primary ferroic ordering - multiferroic materials - have intensified with the aim of exploring novel and interesting features, like multicaloric effect and magnetoelectric (ME) coupling^1,2^. Through a theoretical approach, Vopson demonstrated that multiferroic systems fulfil the requirements to present giant caloric effects which rises from the coupling between its intrinsic ferroic orderings^3^. Therefore such systems have a great potential in magnetic refrigeration technology which is at the forefront to substitute the conventional vapour compression cooling/heating technology. Experimental studies have revealed that the use of multiple-stimuli during each cycle can improve the system caloric efficiency and device operation^4–6^. However, a complete understanding on the interplay between phase transitions on reversible caloric effects of multiferroic materials is still at an early stage^7^.

The ME-coupling in hybrid systems is a product effect which is known to be stronger than in single-phase multiferroic materials^8^. For composites, the ME-coupling rises from interfacial interactions between a piezoelectric and a magnetostrictive phase^9^. Extensive research on these multiple-phase systems revealed that their ME response is strongly dependent on the shape, composition and the connectivity type between the components^10,11^. Among the different designs of multiphase systems presenting large ME responses, it is possible to mention the particulate-matrix composites, bulk and fibers/rods/wires with different shapes and geometries^8^. This feature allows to tune the composite features by choosing the correct components for practical applications. For instance, ME composites can be used as high sensitive magnetic sensors, current/voltage converters and energy harvesters^12^. Considering practical applications, the optimization of the ME response at room temperature is a critical issue towards the design of a real-life device.

The aim of this study is to evaluate the morphological, crystallographic, magnetoelectric and magnetocaloric properties of a micrometric composite obtained by the dilution of magnetostrictive $G{d}_{5}(Si,Ge{)}_{4}$ into a piezoelectric poly(vinylidene) fluoride (PVDF) matrix using a simple chemical route. The concentrations of 2 and 12 weight fraction (wt.%) of magnetic material were chosen for being below the percolation threshold while simultaneously allowing to study the effect of changing one order of magnitude in the magnetic material amount inside the electroactive polymer. Gadolinium silicides germanides have already shown their potential for applications on multi-energy conversion and energy harvesting when implemented into polymeric matrix^13–15^. Ozaydin and Liang demonstrated that the energy conversion from magnetic energy to electrical power is more effective for the crushed Gd${}_{5}$Si${}_{2}$Ge${}_{2}$ alloy blended with PVDF than for pure PVDF and magnetic material itself^13^. More recently, Harstad *et al*. evaluated the enhancement in the generation of voltage output through mechanical stimulation of PVDF loaded with small amounts ($\le$5 wt.%) of 470 nm Gd${}_{5}$Si${}_{4}$ particles^14^. The tests performed by the authors revealed a power density of 14.3 mW/cm${}^{3}$, being more efficient than pure PVDF that presented 3.25 mW/cm${}^{3}$ in the same experimental conditions. These important observations are a consequence of the ME-coupling in composites with magnetostrictive and piezoelectric phases. The evaluation of the ME behavior can be used to improve its response aiming future applications^12^. For instance, the combination of magnetic and electric fields in FeRh thin film deposited into a BaTiO${}_{3}$ substrate revealed a 96% reduction on the magnetic hysteresis losses arising from the strong magnetoelectric coupling^2^. From first principles calculations, it was shown that the ME-coupling for multiferroic systems can be enhanced indirectly by the pyroelectric and magnetocaloric features of the material^16^. In this sense, the results and conclusions presented herein demonstrate the effect of PVDF pyroelectricity on the Gd${}_{5}$(Si,Ge)${}_{4}$ magnetocaloric effect leading to the observation of a multicaloric effect; thus, enabling the advancement of device engineering^1,17,18^.

## Results

**Figure 1:**
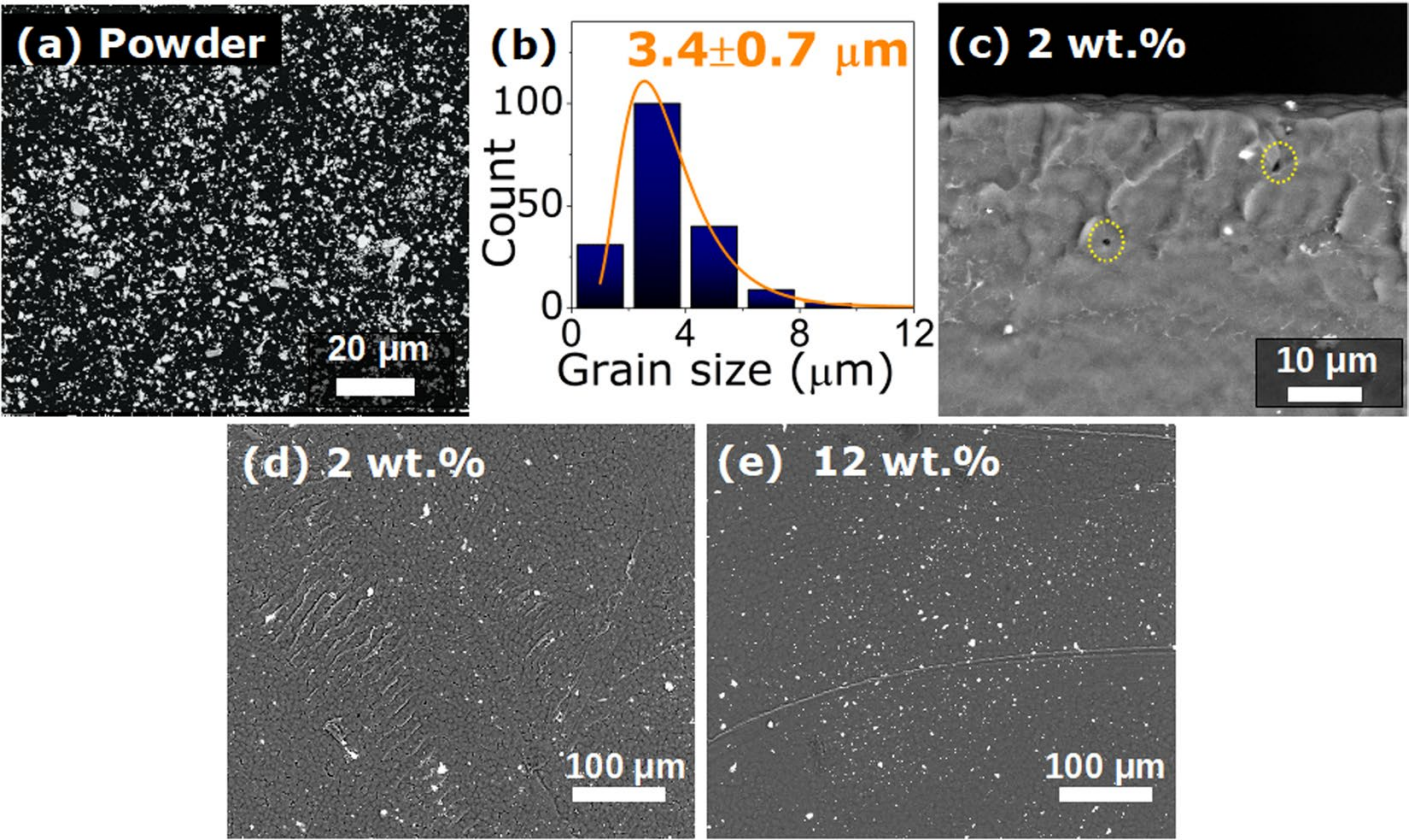
SEM micrographs for (**a**) Gd${}_{5}$Si${}_{2.4}$Ge${}_{1.6}$ powder with average particle size of 3.4 $\mu$m, as obtained through the log-normal distribution shown in (**b**). The cross-section micrograph for 2 wt.% sample in (**c**) reveals the powder distribution along PVDF thickness. **(d)** 2 wt.% and (**e**) 12 wt.% PVDF/GSG composite surface images reveal a good dispersion of the 3.4 $\mu$m Gd${}_{5}$Si${}_{2.4}$Ge${}_{1.6}$ powder.

The SEM micrograph shown in Fig. 1 revealed that, by sieving the Gd${}_{5}$Si${}_{2.4}$Ge${}_{1.6}$ (GSG) milled ingot, a thin powder with an average particle size of 3.4 $\pm$ 0.7 $\mu$m is achieved, as obtained through the histogram of Fig. 1(b). These microparticles were blended with 2 and 12 wt.% concentration into PVDF through solvent casting technique. From the cross-section image of freeze-fractured GSG/PVDF composite, depicted in Fig. 1(c), it is noticeable the well dispersed magnetic microparticles along the polymer volume. As can be seen from Fig. 1(d,e), the polymer morphology does not suffer significant changes when GSG concentration is increased, which is in agreement with the observed for undrawn PVDF membranes^19–21^. Furthermore, from pure PVDF to 12 wt.% composite, the cross-section images reveal that the thickness remains unaltered at around 200 $\mu$m.

**Figure 2:**
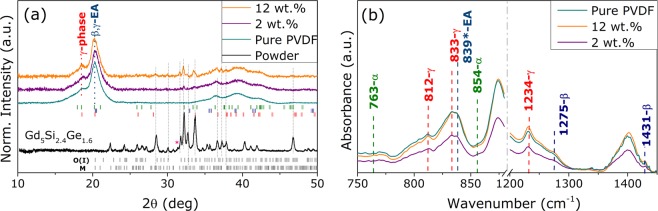
(**a**) XRD patterns of GSG powder, pure PVDF and the composite samples with 2 and 12 wt.%. The main peaks of O(I) and the secondary M structure are highlighted, which are also observed for the composites. The Bragg positions of PVDF crystallographic $\alpha$-, $\beta$- and $\gamma$-type structures are indicated for the composite samples. (**b**) FTIR absorption curves for PVDF and composite samples with the modes of vibration indexed for each $\alpha$-, $\beta$- and $\gamma$-phases.

The XRD patterns obtained for all samples are shown in Fig. 2(a). The GSG powder presents 76.2% of orthorhombic-I [O(I)] and 22.6% of distorted monoclinic (M) structures and less than 2% of eutectic 5:3-phase (Mn${}_{5}$Si${}_{3}$-type), a common product of the fast cooling after melting^22^. Such phase analysis was performed through *Rietveld* calculations and the obtained lattice parameters for O(I), M and 5:3 phases are displayed in the Supplementary Fig. S1, in agreement with Reference ^23^. The main diffraction peak of spurious 5:3 is positioned at 31.2${}^{o}$ corresponding to (210) diffraction plane - identified with an asterisk in Fig. 2(a). As for the pure PVDF and blended systems, the patterns reveal a good crystallinity degree for the polymeric matrix. PVDF is a semi-crystalline plastic formed by C-H-F chains with arrangements mainly observed in three different crystalline structures: $\alpha$-, $\beta$- and $\gamma$-phase^24^. A *LeBail* calculation for pure PVDF pattern was performed and is presented in the Supplementary Fig. S2, revealing a majority formation of electroactive (EA) $\beta$- and $\gamma$-phases. For the composite samples, due to the large amount of crystallographic phases from filler and polymeric matrix and reduced peak intensities, a reliable pattern fitting was not possible to be performed. Furthermore, the absence of additional peaks suggests that there was no contamination during powder manipulation for the composite preparation.

Fourier-transform infrared spectroscopy (FTIR) measurements were performed to quantify the amount of EA-phases in the pure PVDF and composite samples, shown in Fig. 2(b), where the exclusive peak for each crystal structure will be used to distinguish their formation^25^. These peaks are identified in Fig. 2(b) for the three main phases $\alpha$, $\beta$ and $\gamma$ at 763 cm${}^{-1}$, 1275cm${}^{-1}$ and 1234cm${}^{-1}$, respectively, confirming the XRD analysis on the formation of these C-H-F chains for all samples.

**Figure 3:**
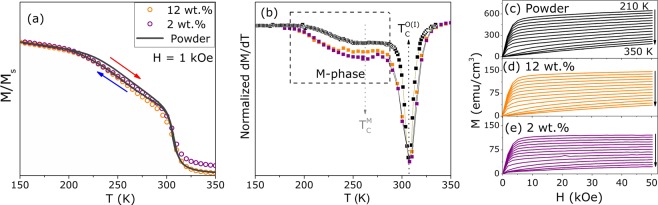
(**a**) Normalized magnetization $M/{M}_{S}$ curves as a function of temperature and (**b**) its temperature derivative curves with the indication on the M-phase transition region and the O(I)-phase Curie temperature. Magnetization isotherms curves for (**c**) 2 wt.%, (**d**) 12 wt.% PVDF/GSG composites and (**e**) for the powder.

The normalized magnetization $M/{M}_{S}$ curves as a function of temperature, for low values of applied magnetic fields, are shown in Fig. 3(a). The thermal hysteresis between cooling (blue arrow) and heating (red arrow) M-T curves for the GSG starting powder is an evidence of a first order magnetic transition (FOMT) attributed to the M-phase of the Gd${}_{5}$(Si,Ge)${}_{4}$ compounds family^26^. This is translated into a bump on the temperature derivative curves of magnetization for the powder and for the composite samples, as highlighted in Fig. 3(b). When the powder is implemented into PVDF, the M-phase transition is conserved with the same T${}_{C}$ around 254 K. The ferro- to paramagnetic (FM-PM) transition of the O(I)-phase occurs at 308 K and is in accordance with previous reports on this composition^23,27^. Figure 3(c–e) shows the M(T,H) map for each sample, focusing on the evaluation of the magnetocaloric properties. As observed for magnetic composites, the dilution of the ferromagnetic powder in the diamagnetic polymer matrix leads to a reduction in the saturation magnetization values^28^.

**Figure 4:**
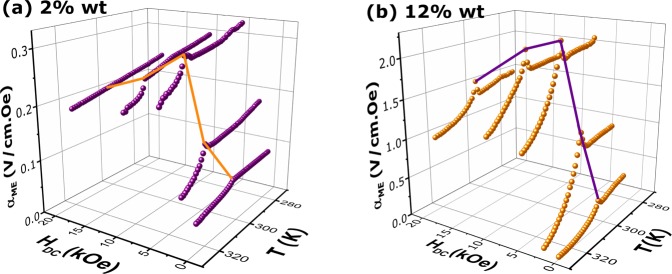
ME voltage coefficient ${\alpha }_{ME}$ as a function of temperature for (**a**) 2wt.% and (**b**) 12 wt.% PVDF/GSG composite samples at different DC magnetic fields. The maximum of ${\alpha }_{ME}$ occurs around 305 K, which is close to FM-PM transition, as observed in the magnetic analysis.

The temperature dependence of ME coefficient for 2 wt.% and 12 wt.% PVDF/GSG composite samples is presented in Fig. 4(a,b), respectively. A maximum in the ME coefficient is observed around $\sim$305 K which shifts by increasing ${H}_{DC}$ bias magnetic field. As can be noted, from 2 to 12 wt.% of filler content, there is a 10$\times$ enhancement in the ME coefficient. These maxima in the ${\alpha }_{ME}$-T curves can be attributed to strong magnetoelectric ordering near ${T}_{C}$. In Fig 4, by increasing ${H}_{DC}$ bias magnetic field, the ME effect increases and reaches the maximum value at a bias field of 5 kOe; then the ME coefficient decreases slowly as the magnetic ${H}_{DC}$ increases. The ME-coupling is closely related to the magnetostrictive behavior of the GSG component^26^. The initial gain in ME coefficient can be attributed to the enhancement of the domain wall movement and the rotation of GSG particles, which facilitates the magnetostriction in the GSG phase. When the bias field approaches 10 kOe, the magnetic field induced strain in GSG micropowders begin to reach saturation. As a result, above 5 kOe, the ME output voltage generated from the mechanical interaction between the phases decreases.

## Discussion

As aforementioned, the coupling between the magnetic and electric orderings of a multicomponent system rises from interfacial interactions^8^. For this reason, the morphology of the produced samples was first analysed through microscope imaging, where the formation of pores for the composites reveals that PVDF nucleates around the particles into a complex structure, with formation of air gaps^19,29,30^. The observed surface profile for the pure PVDF films, however, is in good agreement with previous systems submitted to thermal treatments to improve $\beta$-phase formation with lower porosity levels^31^. This evidence can be related to the chemical ratio quantities and the temperature control during the solvent evaporation^32^.

**Figure 5:**
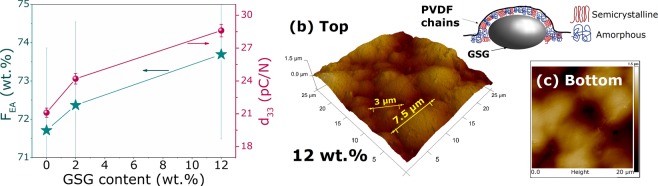
(**a**) (*left axes*) The amount of electroactive phases obtained through the FTIR measurements using Eq. 1 and (*right axes*) the d${}_{33}$ piezoelectric coefficient measured for pure PVDF and composites. Atomic force microscopy for 12 wt.% composite obtained at both sides (**b**) top and (**c**) bottom revealing that PVDF chains nucleates around the magnetic grains.

In this regard, it is important to evaluate the crystallographic and magnetic features of the constituents. The structural characterization of the magnetic filler revealed the formation of magnetostrictive M-phase on the 3.4 $\mu$m powder, that is required for the ME-coupling. As for the electroactive polymer, the XRD analysis has shown a majority formation of EA-phases, responsible for the piezo- and pyroelectric effects of PVDF. However, since PVDF usually presents $\sim$50% of crystallinity degree^21^, the amount of EA-phase formation will be inferred from the FTIR measurements presented in Fig. 2(b). This can be performed by considering the exclusive peaks of $\alpha$-phase at 763 cm${}^{-1}$ and the common peak associated with the vibration modes of the EA-phases, namely, $\beta$ and $\gamma$, placed at 839cm${}^{-1}$^25^. The quantification of EA-phase is then calculated using the relative intensities through the following equation^25^:

1 $${F}_{EA}=\frac{{I}_{EA}}{({K}_{840}/{K}_{763}){I}_{763}+{I}_{EA}}$$

where ${I}_{EA}$ and ${I}_{763}$ are the absorbance intensity of each phase. The constants ${K}_{763}$ and ${K}_{840}$ are related to the absorption coefficients associated with each wavenumber with values of 7.7$\times$10${}^{-4}$ cm${}^{2}$mol${}^{-1}$ and 6.1$\times$10${}^{-4}$ cm${}^{2}$mol${}^{-1}$, respectively^25^. The obtained results are shown in Fig. 5(a) revealing a slight increase in the amount of EA-phases from pure PVDF to 12 wt.% GSG/PVDF composite, corroborating XRD analysis. The enhancement of EA-phases formation and stabilization on PVDF due to the presence of fillers has been reported previously^14,19,20,24,33^. The piezoelectric ${d}_{33}$ coefficient for the pure PVDF and composite samples is also depicted in Fig. 5(a), revealing the same increasing trend of the electroactive phases in the material. We observe therefore an enhancement of the piezoelectric response by increasing the filler content, in agreement with previous reports^14,30,32^. Such observations will be important for the evaluation of the ME-coupling on the produced multiferroic samples.

It is important to highlight that the pyroelectricity of PVDF - where the temperature change leads to polarization variations - affects the caloric response of the composite^34^. In fact, from the morphology evaluation, it was observed the formation of air gaps around the grains that, for MCE applications, favours the heat exchange during machine operation^35,36^. Despite the formation of porous, there is a good bonding between grains and matrix; however, due to the different scattering of each component, it is not possible to ensure a complete covering of the fillers. In order to observe the connections between the grains and the matrix, atomic force microscopy (AFM) measurements were carried out, shown in Fig. 5(b,c). The obtained 3D map for 12 wt.% composite in a selected area of 25 × 25 $\mu$m${}^{2}$ shows a continuous view over the polymer surface where the magnetic particles are covered by PVDF. Images of the opposite side in Fig. 5(c) confirm that all the particles are completely hidden by the polymer. The larger structures present diameters around $\sim$8 $\mu$m, indicating that the polymer layer surrounding the micropowders must be around $\sim$3 $\mu$m in thickness. As illustrated in Fig. 5, the C-H-F chains of the PVDF arrange around the GSG grains during the cast, suggesting that the presence of a magnetic material is influencing the polymer matrix nucleation kinetics^24,30^. This fact will be of great matter for the ME-coupling, where the electric polarization in the piezoelectric phase is driven by the grains displacement during magnetization^8^.

**Figure 6:**
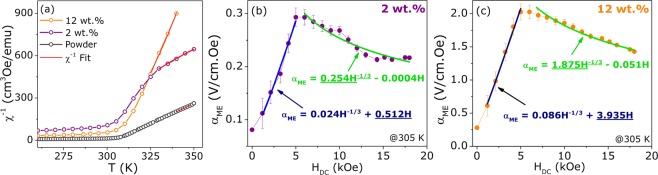
(**a**) Reciprocal magnetic susceptibility curves for the free powder and composite samples with the best fittings of the modified Curie-Weiss law by considering the contribution of each crystallographic structure. The ME voltage coefficient ${\alpha }_{ME}$ as a function of DC magnetic field for (**b**) 2 wt.% and (**c**) 12wt.% PVDF/GSG composite samples at 305 K with the fitted curve considering the thermally mediated mechanism on the ME coupling given by Eq. 6.

Concerning the magnetic behavior, there is no shift in ${T}_{C}$ of the powder for the 2 wt.% and 12 wt.% composites, as observed for other blended systems, revealing no major influence from the ferroelectric polymer on the filler intrinsic magnetic features^13,14,37^. The Gd${}_{5}$(Si,Ge)${}_{4}$ family compounds have a strong magnetic and structural coupling and, for this reason, magnetic analysis can be used to infer the amount of each phase^22,38^. This was performed through the reciprocal magnetic susceptibility curves, considering the contribution of each phase - as detailed in the Supplementary Information Document, based on the References^22,38^. The best fittings to the data are presented in Fig. 6(a) and the corresponding free parameters listed in Table 1. As can be noted from the reciprocal magnetic susceptibility (${\chi }^{-1}$), the diamagnetic contribution of PVDF (${\chi }_{0}$) is more evident for the sample with lower GSG content, due to the ferromagnetic dilution. The ${\theta }_{P}$ values for the main phases in Table 1 suffer a slight reduction from powder to 2 wt.% composite, because of the grains dilution along the polymeric chain. It is worth pointing out that the glass transition temperature (T${}_{g}$) for the amorphous phase of PVDF is around 233 K, where the matrix deformations during melting must be affecting the system magnetic response^39^. PVDF is a diamagnetic material; however, the $\beta$-phase presents a net nonzero dipole moment which can interact with the embedded particles that can be the responsible for the associated errors of the fit parameters^40^. Furthermore, the paramagnetic effective moment (${\mu }_{eff}$) values - obtained through the relation $C={\mu }_{eff}^{2}/3{k}_{B}$ - are within the error for the theoretical expected for Gd${}^{3+}$ for the main O(I)-phase and for reported values on 5:3 binary phase^22,35,41^. Gd${}_{5}$(Si,Ge)${}_{4}$ compositions with a M-structure present high sensitivity to external parameters and, for this reason, there is a reduction in the ${\mu }_{eff}$ values that can be an effect of PVDF walls on the grains surface during the FM-PM transition^37^. Nevertheless, from the data obtained at low temperature (5 K), we could extract the saturation magnetization (${\mu }_{sat}$) values by extrapolating the M *versus* 1/H curve. For the O(I)-phase, the ${\mu }_{sat}$ value is expected to be 7.41 ${\mu }_{B}$ due to the extra contribution from 5d orbitals of Si and Ge^38^; however, due to the formation of M and 5:3 phase there is a reduction in ${\mu }_{sat}$ for the powder. The ${\mu }_{sat}$ lower values for the composite samples are a result of magnetic material dilution that can generate dipolar interactions between the smaller grains inside the electroactive polymer^40,42^.

**Table 1:** Suitable parameters extracted from the magnetic results for Gd${}_{5}$Si${}_{2.4}$Ge${}_{1.6}$ (GSG) powder and GSG/PVDF composites: Curie temperature (${T}_{C}$), paramagnetic Curie temperature (${\theta }_{P}$) and effective moment (${\mu }_{eff}$), saturation magnetization obtained at 5 K (${\mu }_{sat}$), ${\chi }_{0}$ extracted from the susceptibility fittings and the Landau coefficient ${B}_{Landau}$ estimated from the Arrott plot curves.

	Phase wt.%	T${}_{C}$ (K)	${\theta }_{P}$ (K)	${\mu }_{eff}$ (${\mu }_{B}/G{d}^{3+}$)	${\mu }_{sat}$ (${\mu }_{B}/G{d}^{3+}$)	${\chi }_{0}$ (emu/g.Oe)	${B}_{Landau}$ (10${}^{-6}$Oe.FU${}^{3}$/${\mu }_{B}^{3}$)
2 wt.%	O(I) 76.2%	308(5)	293(4)	7.92(2)	6.87(1)	-7.30(4)	6.31(3)
	M 21.9%	254(5)	276(3)	7.43(6)			
	5:3 1.89%	—	184(9)	8.13(5)			
12 wt.%	O(I) 76.0%	308(5)	305(9)	7.92(2)	6.91(1)	-7.88(5)	2.00(1)
	M 21.3%	254(5)	287(8)	7.51(7)			
	5:3 2.74%	—	187(6)	8.11(2)			
Powder 3.4 $\mu$m (100%)	O(I) 76.2%	308(5)	310(4)	7.86(5)	7.00(9)	—	1.39(3)
	M 21.4%	254(5)	293(3)	7.48(7)			
	5:3 2.44%	—	186(4)	8.12(6)			

The ME effect in a 0-3 type multiferroic composite is strongly dependent on the connection between magnetostrictive and piezoelectric components. Some interesting theoretical and experimental results were obtained in Reference^9^ for multiferroic PVDF based composite spheres, where it was concluded that particles with 1.4 $\mu$m size have higher ME-coupling. In this study, since the GSG particles have random shapes, the described models cannot be applied for a clear explanation of interfacial effects. Therewith, it should be noted that our magnetic component, Gd${}_{5}$Si${}_{2.4}$Ge${}_{1.6}$, is a material with large magnetocaloric effect around the Curie temperature that can also contribute to the total ME effect^34^. From first-principle calculations, it was demonstrated that the intrinsic thermodynamic features of a hybrid system can be used to tune the ME-coupling^16^. To understand this mechanism, we should first write the ME-coupling in terms of the adiabatic temperature change $\Delta T$ - from the magnetocaloric effect - and the temperature, as follows:

2 $${\alpha }_{ME}=\frac{dE}{dH}=\left(\frac{\partial E}{\partial T}\right)\left(\frac{\partial T}{\partial \Delta T}\right)\left(\frac{\partial \Delta T}{\partial H}\right)$$

For the GSG magnetocaloric material, the adiabatic temperature change ($\Delta T$) can be given by the Belov-Goryaga equation^34,43^:

3 $$H=\frac{{a}_{1}}{{k}^{1/2}}\Delta {T}^{1/2}+\frac{{a}_{2}}{{k}^{3/2}}\Delta {T}^{3/2},$$

where the phenomenological coefficients are $${a}_{1}={a}_{T}({T}_{C}-T)$$ and *a*_2_, $$k={a}_{T}T/C$$, with *a*_*T*_ being a temperature independent constant and $$C$$, the heat capacity. Thus, at low DC applied magnetic field regime, the dominant term is $$\Delta T\propto $$$${H}^{2}$$; and, for high magnetic fields, $${H}^{2/3}$$ is the predominant one^34^. In this way, the main derivative terms considering different regimes of applied DC field regimes can be written as follows:

4 $$\,{\rm{low\; DC\; field\; :}}\,\Delta T\propto {H}^{2}\to \frac{\partial \Delta T}{\partial H}\propto H;$$

5 $$\,{\rm{high\; DC\; field\; :}}\,\Delta T\propto {H}^{2/3}\to \frac{\partial \Delta T}{\partial H}\propto {H}^{-1/3}.$$

Hence, given the thermally mediated mechanism, the ME coefficient can be described by the following relation:

6 $${\alpha }_{ME}={c}_{1}{H}^{-1/3}+{c}_{2}H,$$

with ${c}_{1}$ and ${c}_{2}$ being the constants related to the two first derivative terms in Eq. 2.

To confirm this behavior, a curve fit on the field dependence of ${\alpha }_{ME}$ was performed considering the above function, that is shown in Fig. 6(b,c) for the 2 wt.% and 12 wt.% composites, respectively. As can be noted, the higher coefficient below the saturation is associated with the linear term (${c}_{2}$) and at the high DC field regime, the ${c}_{1}$ parameter that is related to the power term of Eq. 6 has the larger value, confirming the mechanism description given above. Finally, with these phenomenological observations, it should be concluded that the ME effect in PVDF/GSG composites is a result of the elastic cooperation between magnetostrictive and piezoelectric components behavior with a contribution from a thermal mediation arising from the components magnetocaloric and pyroelectric features.

**Figure 7:**
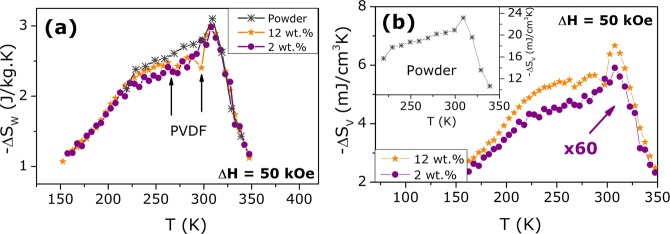
Magnetic entropy change curves for the powder and composite samples calculated (**a**) considering the mass of magnetic material and (**b**) the volume of the measured system with the density values of 1.70 g/cm${}^{3}$ and 1.85 g/cm${}^{3}$, for 2 and 12 wt.% composite samples, respectively.

The adiabatic entropy change was calculated with the M(T,H) maps using the Maxwell-relation $\partial M/\partial T=\partial S/\partial H$, due to the absence of irreversibility between the magnetization curves by increasing and decreasing the applied magnetic field^23,27,44,45^. Therewith, the $\Delta {S}_{M}(T)$ curves for all samples, considering the weight fraction of magnetic material and for a field variation of 5 T, could be obtained as shown in Fig. 7(a). The maximum values ($\Delta {S}_{M}^{max}$) range from 2.88 to 3.10 J/kg.K from powder to 2 wt.% composite, indicating no effect of PVDF matrix on the FM-PM transition at 308 K. However, the $\Delta S$ curve profile suffers a drastic change in the [230–295]K temperature range: for the powder, there is a lump due to the thermal hysteresis of M-phase; 12 wt.% composite shows a deviation from the powder curve at 265 K and 2 wt.% curve has a linear growth above 230 K. Such differences strongly suggest that indeed there is a coupling between the ferroelectric phases of PVDF and the magnetic phase of GSG, which enlarges the ME-coupling presented in the previous section. The glass transition of amorphous $\alpha$-phase occurs around 220 K, that leads to relaxation of semicrystalline chains with a small associated pyroelectric effect that vanishes around 275 K^34,46^. For 2 wt.% sample, due to the low amount of filler, the effect is nearly null at the 200-300 K temperature range. With higher filler contents, however, the pyroelectricity contribution to the $\Delta S$ curve becomes more evident, implying the interaction between the electroactive PVDF and the grains^8,34^. For engineering applications, the most effective unit for the change in entropy is the volumetric one $\Delta {S}_{V}$^47^, depicted in Fig. 7(b). The $\Delta {S}_{V}^{max}$ values naturally decrease from $\sim$24 mJ/cm${}^{3}$K for the starting powder to $\sim$6.62 mJ/cm^3^ K and 0.10 mJ/cm^3^ K when implemented into PVDF with 12 and 2 wt.%, respectively. Nevertheless, the relative cooling power - which is a more effective parameter for device implementation - calculated at the 220–330K temperature interval is 9.50 mJ/cm${}^{3}$ and 638 mJ/cm${}^{3}$ for the 2 and 12 wt.% composites, respectively.

The combination of materials with multiple caloric effects, the so-called multicaloric materials, is a new topic of research for improving alternative cooling technologies^1^. As pointed out by Vopson, systems presenting two primary ferroic orderings simultaneously, the multiferroic materials, are the main candidates to present giant caloric effects^3^. Among the required conditions, the materials must present low heat capacity, large ME-coupling coefficient and low magnetic/electric hysteresis. For the present case, the ${\alpha }_{ME}$ of 2.2 V/cm.Oe obtained for 12 wt.% composite is among the largest ME responses when compared with other 0-3 type composites reported on literature^48^, which is affecting the system MCE response. In this way, the cross-coupling effect should be taken into account for the isothermal entropy change of the system under the influence of an applied magnetic field. The alignment of magnetic particles along the polymeric matrix induces an electric polarization (${P}_{ind}$) and, consequently, an internal electrical field (${E}_{ind}$). If we assume a linear ME-effect, the induced field in the system is^3^:

7 $$d{E}_{ind}=\frac{{\alpha }_{ME}}{{\epsilon }_{0}{\chi }^{e}}dH,$$

with ${\epsilon }_{0}$ being the vacuum permittivity and ${\chi }^{e}$, the electrical susceptibility. In this way, the total entropy change of the multicomponent system, when a magnetic field is applied, derived from the generalized Maxwell relations in Reference^3^, is given as follows:

8 $$\Delta {S}_{total}={\int }_{\Delta H}{\left(\frac{\partial M}{\partial T}\right)}_{{E}_{ind}}dH+{\int }_{\Delta H}\frac{{\alpha }_{ME}}{{\epsilon }_{0}{\chi }^{e}}{\left(\frac{\partial {P}_{ind}}{\partial T}\right)}_{H}dH.$$

The curves presented in Fig. 7 were indirectly obtained through the first term of Eq. 8, that can be simply denoted as $\Delta {S}_{M}$. Rearranging this relation for the calculated $\Delta {S}_{M}$ we obtain:

9 $$\Delta {S}_{M}=\Delta {S}_{total}-{\int }_{\Delta H}\frac{{\alpha }_{ME}}{{\epsilon }_{0}{\chi }^{e}}{\left(\frac{\partial {P}_{ind}}{\partial T}\right)}_{H}dH$$

The polarization derivative term represents the pyroelectric effect that, in the present system, rises from the PVDF electroactive phase relaxation^3,46^. Hence, the polymer ferroelectricity is contributing to the magnetic entropy change which justifies the deviation from the powder curve observed around 270 K for 12 wt.% composite. It is important to point out the dependence on the ME coefficient, which is also affecting the composite caloric behavior. For this reason, the understanding on the mechanisms between each phase in a multiferroic composite system is relevant from a fundamental point of view aiming prototype development. In this sense, in order to completely understand the mechanism behind the coupling between magnetic and electric phase orderings on the multicomponent system, an evaluation of the electrocaloric response of the samples should be performed^2^. Nevertheless, these results reveal that the cross-coupling effects play a role on the MCE behaviour of GSG/PVDF composites which can be used to tune these features for future applications^3,28,40^.

## Conclusions

In this study we have experimentally demonstrated that the introduction of Gd${}_{5}$Si${}_{2.4}$Ge${}_{1.6}$ microparticles into an electroactive PVDF with a volume fraction of 2 and 12 wt.% gives rise to a magnetoelectric coupling and, consequently, a multicaloric effect. Morphological and structural characterization revealed an improvement in the polar $\beta$- and $\gamma$-phases on the PVDF due to the magnetic filler, that is responsible for the piezo- and pyroelectric effects. As a result, a large ME response of 2.2 V/cm.Oe is observed for 12 wt.% of powder concentration. Similarly, this strong coupling between magnetic and electric orderings on the produced composites lead to unexpected variations in the magnetic entropy change curves when compared with the pure magnetic material. The change of the powder magnetocaloric response can be attributed to the pyroelectricity character of the polymer, where the increase of temperature induces polarization variations in the polymeric matrix^3^. These results reveal the great potential of Gd${}_{5}$(Si,Ge)${}_{4}$ family for applications as sensors and energy harvesting by the combination with polymeric matrices even with low magnetic material concentration.

## Methods

### Samples production

Tri-arc melting technique was used for the preparation of magnetic material with Gd${}_{5}$Si${}_{2.4}$Ge${}_{1.6}$ (GSG) stoichiometry, as described in Reference^27^. The microparticles were produced by sifting the as-cast ingot powder through a sequence of strainers with hole sizes from 50 $\mu$m to 5 $\mu$m. The obtained powder was blended with poly(vinylidene) fluoride (PVDF) using the solvent casting technique. For this procedure, PVDF powder (acquired from Alfa Aesar, 44080) was dissolved in dimethylformamide (DMF) (from Sigma, 227056-1L) with a weight ratio of 1:30 in a hot plate at 310 K to obtain a final solution with a volume of 1 ml. The amount of magnetic material dispersed on the suspension was weighted with 2% and 12% weight fractions of PVDF. Subsequently, the solution dried for a day in exhaust hood.

### Characterization techniques

SEM micrographs were carried using a Phyllips-FEI/Quanta 400 with 500-10 000$\times$ magnification with an energy of 15 kV at Material Centre of Porto University (CEMUP). For cross-section imaging, all the composite films were fractured after being frozen in liquid Nitrogen. Structural characterization was performed by means of X-ray Diffraction (XRD) at room temperature using a Rigaku Smartlab with a Cu-K$\alpha$ radiation, 45 kV and 200 mA at IFIMUP and analyzed using the *FullProf Suite Software*^49^. The amount of each crystal phases on the polymorphic PVDF film was obtained through FTIR measurement. Data were collected in the range of 550-1400 cm${}^{-1}$ at room temperature using a Jasco Deutschland, (Model FT/IR-6100 type A) spectrometer at the absorption mode with a 2 cm${}^{-1}$ resolution at LAMULT-Unicamp. The piezoelectric coefficient measurements were carried out using a d${}_{33}$ meter (Model YE2730). All samples were coated with Ag contacts on both sides and were polled by applying an electric field of 20 kV/mm for 1 hour at 50 °C. During poling, the samples were placed in an oil bath with a thermo controller. Magnetic characterization and MCE evaluation were performed using a Superconducting Quantum Interference Device (SQUID) Magnetometer with data collected within the range of [5,350] K under applied magnetic fields up to 5 T at IFIMUP facility.

### Magnetoelectric (ME) measurements

Using a custom designed setup in a 77-350 K temperature range at Amirkhanov Institute of Physics, Daghestan Scientific Center. ME effect was studied by measuring a voltage U generated across the sample under superimposed alternating magnetic field ${H}_{AC}$ and static bias magnetic field ${H}_{DC}$, as described in Reference^50^. The ME signal was measured by a lock-in amplifier (Stanford research system, Model SR830) and AC magnetic field was generated by internal waveform generator of SR830. The amplitude of AC magnetic field was $\sim$10 Oe with 50–70 kHz of frequency, as pointed in Fig. S3, and DC magnetic field was applied in 0-18 kOe range. The ME coefficient ${\alpha }_{ME}$ is defined using the relation:

10 $${\alpha }_{ME}=\frac{dE}{dH}=\frac{dU}{b.dH}=\frac{{U}_{AC}}{b.{H}_{AC}}$$

where $${U}_{AC}$$ is the magnetically induced AC output voltage across the plane of the sample, $${H}_{AC}$$ is the amplitude of the AC magnetic field and $$b$$ is the sample thickness. ME coefficient was measured in mode, where the applied bias magnetic field $${H}_{DC}$$ is parallel to the direction of ME voltage ($${H}_{DC}$$$$\parallel $$ U) and perpendicular to the plane of sample. The samples used for magnetoelectric measurements have the shape of thin rectangular plates with sizes of 0.26 $$\times $$4 $$\times $$ 7 mm and 0.17 $$\times $$ 4 $$\times $$ 3 mm, for 2 wt.% and 12 wt.% composites, respectively.

## Supplementary information

Supplementary Information

## Data Availability

All data generated or analysed during this study are included in this published article (and its Supplementary Information files).
