# Supplementary material for: Multicaloric effect in a multiferroic composite of Gd5(Si,Ge)4 microparticles embedded into a ferroelectric PVDF matrix
Source: Sci Rep. 2019 Dec 4;9:18308. doi: 10.1038/s41598-019-54635-8 (PMC6892869; doi:10.1038/s41598-019-54635-8)
Supplement: Supplementary file 1 — Supplementary Information [file 41598_2019_54635_MOESM1_ESM.pdf]

Supplementary Information

Multiferroic Composite as a multifunctional magnetocaloric material: $Gd_5Si_{2.4}Ge_{1.6}$ microparticles embedded into a ferroelectric PVDF matrix

Vivian Andrade[1,2], Abdulkarim Amirov[3,4], Dibir Yusupov[4], Bruno Pimentel[5], Nathalie Barroca[1], Ana Pires[1], João Belo[1], André Pereira[1], Manuel Valente[6], João Araújo[1], Mario Reis[5]

[1] IFIMUP and IN-Institute of Nanoscience and Nanotechnology, Physics and Astronomy Department of Science Faculty, University of Porto, Rua do Campo Alegre, 687, 4169-007 Porto, Portugal.

[2] 'Gleb Wataghin' Physics Institute, Campinas State University (UNICAMP), C. P. 6165, 13.083-970 Campinas S.P., Brazil

[3] Laboratory of Novel Magnetic Materials & Institute of Physics Mathematics and Informational Technologies, Immanuel Kant Baltic Federal University, Kaliningrad, Russia.

[4] Amirkhanov Institute of Physics Daghestan Scientific Center, Russian Academy of Sciences, Makhachkala, Russia.

[5] Physics Institute, Fluminense Federal University, Av. Gal. Milton Tavares de Souza s/n, 24210-346, Niteroi-RJ, Brasil.

[6] Department of Physics and I3N, University of Aveiro, 3810-193, Aveiro, Portugal

***** Correspondence and requests for materials should be addressed to Mario Reis (e-mail: marior@if.uff.br)

Rietveld refinement $Gd_5Si_{2.4}Ge_{1.6}$ bulk and micrometric powder

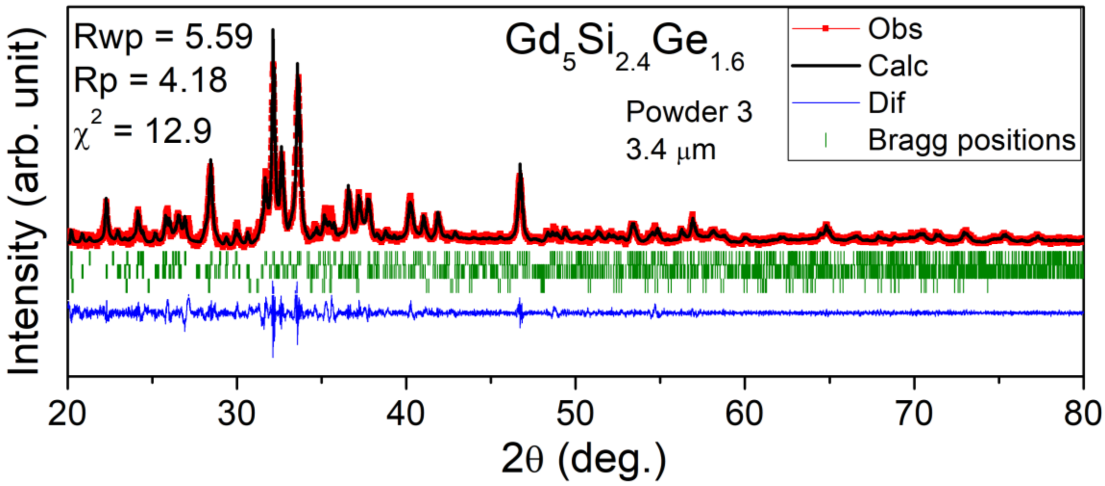

Figure S 1 *Rietveld calculation and quality of fit parameters for the pattern obtained for* $Gd_5Si_{2.4}Ge_{1.6}$ *powder with 3.4 µm using a Rigaku Smartlab Diffractometer with a Cu-Kalpha source. Three structures were identified through Rietveld refinements using Fullproff Suite software[1]: orthorhombic-I* Gd_5Si_4*-type (Pnma space group) with the lattice parameters a = 7.54 Å, b = 14.8 Å, c = 7.80 Å and V = 869 Å*^{3}*, corresponding to a fraction of 76.2%; 23.4% of monoclinic* $Gd_5Si_2Ge_2$*-type (*$P112_{1/a}$ *space group) with a = 7.50 Å, b = 14.7 Å, c = 7.78 Å, γ = 93.2*^{o} *and V = 858 Å*^{3}*; and 0.44% of eutectic* $Gd_5(Si_{0.6}Ge_{0.4})_3$*-phase (*$P6_3/mcm$ *space group) with a = b = 8.76 Å, c = 6.30 Å and V = 418 Å*^{3}*.*

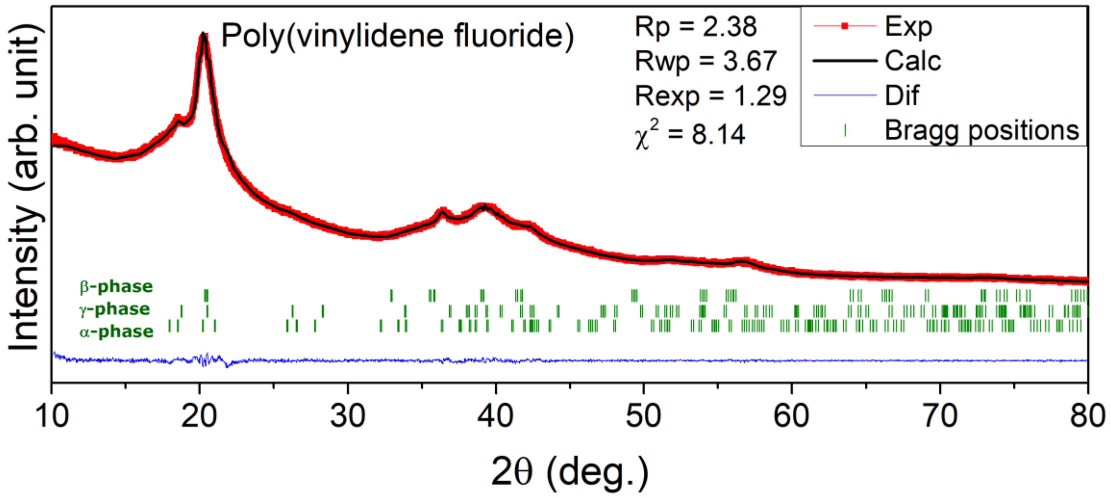

Figure S 2 *LeBail calculations for pure PVDF using the initial lattice parameters from Ref. [1] with the identification of the three phases: α-phase (P 21/c space group) with V = 221.9(5) Å*^{3}*, β-phase (Cm2m space group) with V = 118.1(4) Å*^{3} *and γ-phase (P21/c space group) with V = 123.7(3) Å*^{3}*.*

Magnetoelectric coupling AC field dependence

The magnetoelectric (ME) coupling measurement is performed by considering an AC magnetic field (H_{AC}) in order to reduce the required intensities of DC bias field (H_{DC}). The ME coefficient (α_{ME}) dependence on frequency of modulated H_{AC} magnetic field at zero H_{DC} bias magnetic field at room temperature for 2 and 12 wt.\% PVDF/GSG composite samples are shown in Figure S3. At the frequencies of about 53 kHz and 71 kHz, the resonance-induced enhancement in the ME effect is observed for 2 wt.\% and 12 wt.\% samples, respectively. For the evaluation on the ME-coupling on the produced samples, the AC field are held constant at the higher resonance frequencies to measure the systems output by considering different DC bias magnetic field and temperature.

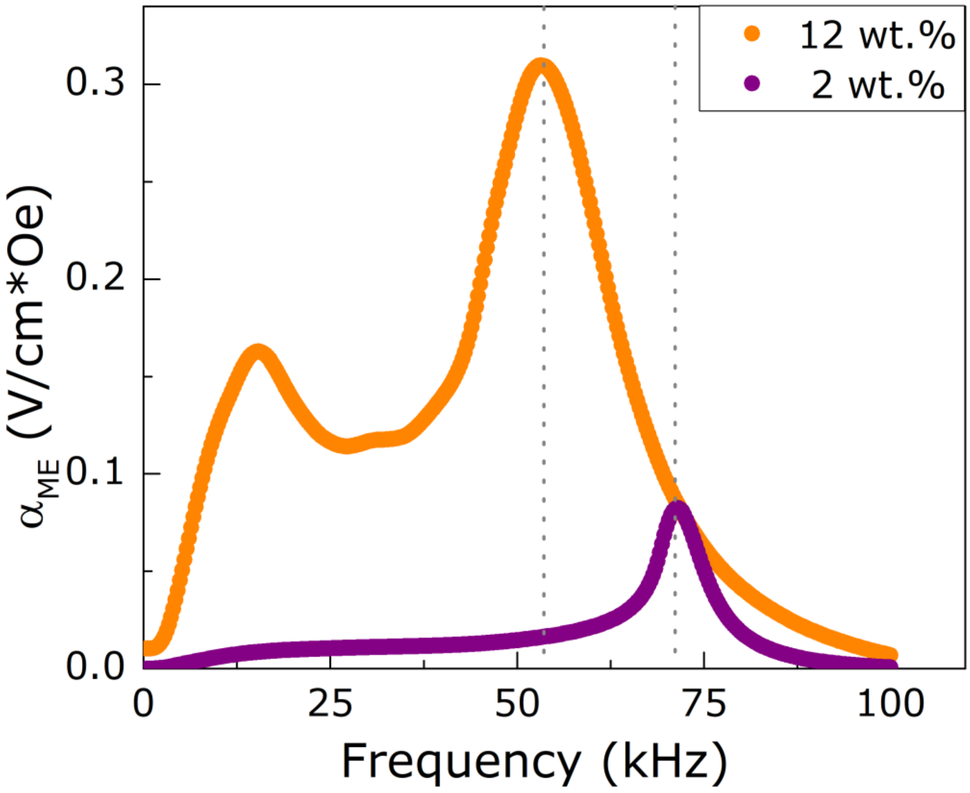

***Figure S 3** Magnetoelectric coefficient as a function of AC magnetic field frequency at room temperature with a null DC bias for the 2 and 12 wt.% GSG/PVDF composite samples.*

Reciprocal Magnetic Susceptibility for a multiple-phase system

From XRD analysis, it was found the formation of three phases: O(I), M and 5:3 structures with the parameters presented above. The magnetic measurements can be used to confirm crystallographic results in the $Gd_5(Si,Ge)_4$ family compounds. In these systems, the magnetic moments are localized with long-range RKKY-type exchange interactions [2]. At the paramagnetic state, there is only non-cooperative interactions

which allows to write the total magnetization of a material by summing up each phase contribution: $\sum_i x_i M_i$. Considering the Curie constant (C_i) and the paramagnetic Curie temperature (θ_P^i) of each crystallographic phase amount (x_i), the magnetic susceptibility can be written as [3,4]:

$$\chi = \frac{x_1 C_1}{T - \theta_P^1} + \frac{x_2 C_2}{T - \theta_P^2} + \ldots + \frac{x_n C_n}{T - \theta_P^n} = \sum_{i=1}^{n} \frac{x_i C_i}{T - \theta_P^i} \qquad (1)$$

The linear region of the reciprocal magnetic susceptibility is given, by definition, at the limit of low applied magnetic fields ($H \rightarrow 0$) and, for this reason, the calculations were performed at the magnetization data obtained under an applied magnetic field of 0.1 T. For the composite samples, it is required to consider the diamagnetic contribution from PVDF (χ_0). The curves fitting was performed using the Levenberg Marquardt Interaction Algorithm at the Nonlinear Curve Fit option of Origin Pro 9.0 software considering the following relation:

$$\chi^{-1} = \frac{1}{\frac{x_{O(I)} C_{O(I)}}{T - \theta_P^{O(I)}} + \frac{x_M C_M}{T - \theta_P^M} + \frac{x_{5:3} C_{5:3}}{T - \theta_P^{5:3}} + \chi_0} \qquad (2)$$

The best calculations are presented in Figure S4, with the parameters summarized in Table S1. It is worth to point out that the amount of each phase was considered to sum up 1 with the magnetization data in CGS units.

Table S 1 Returned parameters from the best calculated reciprocal magnetic susceptibility curves for all samples.

**Parameters**	**Powder 3.4 μm**	**2 wt.% GSG/PVDF**	**12 wt.% GSG/PVDF**
$x_{O(I)}$	0.7620	0.7620	0.7600
x_M	0.2136	0.2191	0.2126
$x_{5:3}$	0.0244	0.0189	0.0274
$C_{O(I)}$	0.05229	0.0530	0.0532
$\theta_P^{O(I)}$ (K)	292.94	293.12	305.01
C_M	0.1650	0.1509	0.1710
θ_P^M (K)	287.24	276.03	286.92
$C_{5:3}$	1.7408	2.1301	1.5460
$\theta_P^{5:3}$ (K)	186.14	184.13	187.20
χ_0 (Oe.g/emu)	-	7.3E-4	7.9E-4

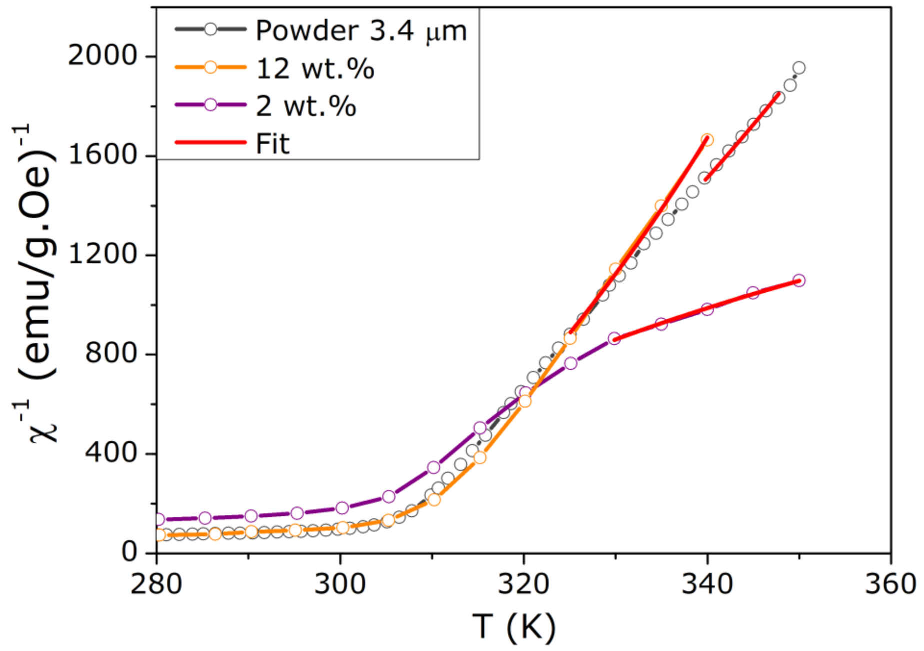

Figure S 4 Best fitted curves for all samples calculated using Eq. (2) with the magnetization data in emu/g. The diamagnetic contribution from PVDF matrix is more evident for 2 wt.% composite due to the change in curvature at higher temperatures.

Arrott plot's curves

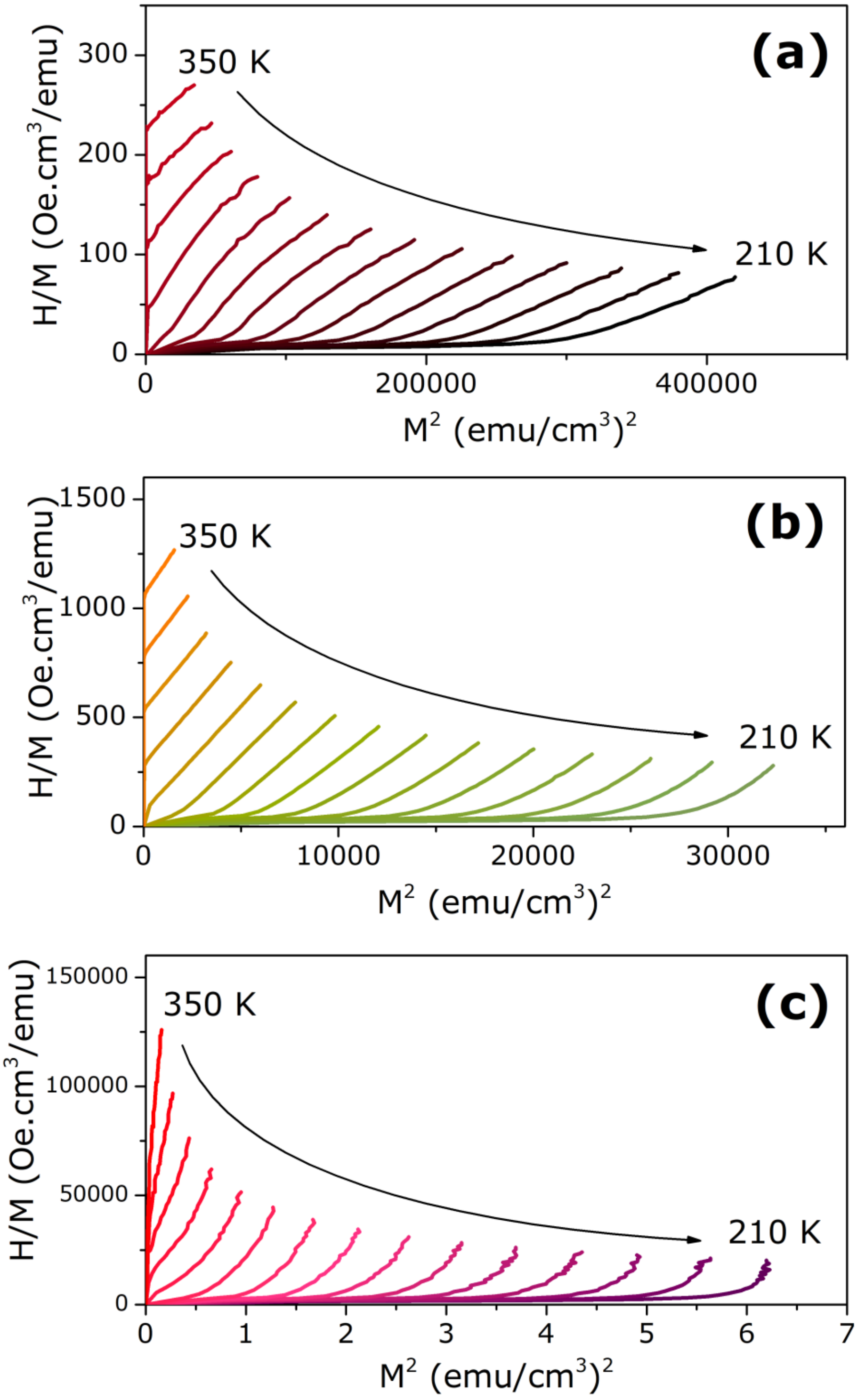

Figure S 5 Arrott plot curves calculated using the M(T,H) map for $Gd_5Si_{2.4}Ge_{1.6}$ (a) powder with 3.4 µm, (b) 2 wt.% and (c) 12 wt.% GSG/PVDF composites revealing a second order magnetic transition, according to the Barnejee criterion.
